# Declines in Reproductive Condition of Male Largemouth Bass (*Micropterus salmoides*) Following Seasonal Exposure to Estrogenic Endocrine-Disrupting Compounds

**DOI:** 10.3390/ijms232416131

**Published:** 2022-12-17

**Authors:** Jessica K. Leet, Catherine A. Richter, Robert W. Gale, Donald E. Tillitt, Jill A. Jenkins

**Affiliations:** 1 U.S. Geological Survey, Columbia Environmental Research Center, Columbia, MO 65201, USA; 2 U.S. Geological Survey, Wetland and Aquatics Research Center, Lafayette, LA 70506, USA

**Keywords:** atrazine, largemouth bass, gonadosomatic index, sperm, recrudescence, ethinylestradiol, estrone

## Abstract

Reproductive abnormalities, that could lead to possible effects at the population level, have been observed in wild fish throughout the United States, with high prevalence in largemouth bass (LMB; *Micropterus salmoides*) and smallmouth bass (*Micropterus dolomieu*). Estrone (E1) and atrazine (ATR) are common environmental contaminants often associated with agricultural land use. 17alpha-ethinylestradiol (EE2) is a contaminant associated with wastewater treatment effluent, and a representative, well-studied estrogen commonly used for fish toxicity testing. Our objective was to assess whether early gonad recrudescence in adult fish was a period of sensitivity for alterations in reproductive condition and function. Adult male LMB were exposed from post-spawning to early gonad recrudescence to either a mixture of E1 (47.9 ng/L) + ATR (5.4 µg/L), or EE2 (2.4 ng/L) in outdoor experimental ponds. Gonad samples were collected from fish just prior to the start of exposure (July), at the end of the exposure period (December), the following spring just prior to spawning (April), and post spawning (May). Gonadosomatic index (GSI) was significantly reduced in E1 + ATR-exposed and EE2-exposed males compared to control at every post-exposure time point. Reduced sperm count and sperm motility were observed in the mixture treatment (E1 + ATR) compared to the control. Sperm motility was also reduced in the EE2 treatment. These data together indicate that estrogenic endocrine-disrupting compounds can lessen the reproductive condition of adult male LMB, and that effects of exposure during early gonad recrudescence can persist at least through the subsequent spawning cycle.

## 1. Introduction

Reproductive abnormalities have been observed in wild fish throughout the United States, with high prevalence in largemouth and smallmouth bass [1,2,3,4]. In the Chesapeake Bay watershed for example, male abnormalities have been highly prevalent in smallmouth bass (*Micropterus dolomieu*), and to a lesser extent largemouth bass (LMB; *Micropterus salmoides*). Abnormalities in male gonads have been correlated with the presence of the agricultural pesticide atrazine (ATR), which is widely used and one of the most commonly detected pesticides in the watershed [5]. Average ATR concentrations can exceed 5 µg/L in areas of high agricultural land use, with spikes in the spring potentially above 20 µg/L [6,7,8,9]. Estrone (E1), a naturally occurring estrogen, has also been found in the majority of water samples analyzed from this watershed [7,8] and associated with reproductive abnormalities in fish [10]. In areas with heavy influence from surrounding agricultural land use, estrone can be found at concentrations that spike above 100 ng/L in the springtime. Such high estrone levels may be of concern for fish reproductive health [11,12,13].

Surface waters influenced by higher urban land use and presence of wastewater treatment plants are often contaminated with 17alpha-ethinylestradiol (EE2) at concentrations below 1.0 ng/L. Concentrations up to 10 ng/L have been observed [14]. These are concentrations that have been shown to impact fish development and reproduction [12,15]. Adult LMB have been shown to be sensitive to induction of reproductive abnormalities in response to chronic exposure to a model estrogen (EE2) over a complete reproductive cycle [16]. However, it is not known at what times during the reproductive cycle bass are most sensitive to exogenous hormone and/or contaminant exposure. LMB have seasonal, relatively synchronous spawning that begins once water temperatures reach 62 °F or above [17]. Late summer to fall is the period of early gonad recrudescence when spermatogenesis and oogenesis are beginning, in preparation for spawning in the following spring [18,19]. Early recrudescence in males is a time of rapidly proliferating spermatogonia and may be sensitive to exogenous stimulatory input [20].

Sperm motility is a conventional andrology parameter that is prognostic for fertilization, and thus reproductive capability [21,22]. Male gamete (sperm cell) quality and quantity (cell count) have been positively correlated with the degree of fertilization success in cultured fish species [23]. However, such rigorous investigations are uncommon for wild fish and popular sport fishes, such as smallmouth bass and LMB within the United States [24].

The extent to which ATR and/or estrogens can influence reproductive condition and function in bass species is unexplored. There have been correlative associations between environmental concentrations of either E1 or ATZ and the incidence of gonadal anomalies and reproductive condition of bass in field studies [5,10]. Therefore, experimental studies of bass exposed to these compounds under controlled conditions are needed to establish causative linkages. Both E1 and ATZ are known endocrine-disrupting compounds with potential to disrupt reproductive homeostasis in either males or females. The focus in the current study was on male reproductive condition and health because correlations of gonad anomalies in field studies were specific to male testes [10]. EE2 was chosen as a positive control because it is commonly used in endocrine disruption studies evaluating potentially estrogenic chemical contaminants [25]. Our objective was to assess whether early gonad recrudescence was a period of sensitivity for alterations in reproductive capability. Adult LMB were exposed in outdoor experimental ponds from post-spawning through early gonad recrudescence to either EE2, or a mixture of E1 + ATR. Gonad samples were collected from fish just prior to the start of exposure (July), at the end of the exposure period (December), the following spring just prior to spawning (April), and post spawning (May). Male reproductive condition was assessed by measuring gonadosomatic index (GSI; all timepoints), as well as sperm cell count and sperm motility (April).

## 2. Results

### 2.1. Exposure Analysis

**Figure 1:**
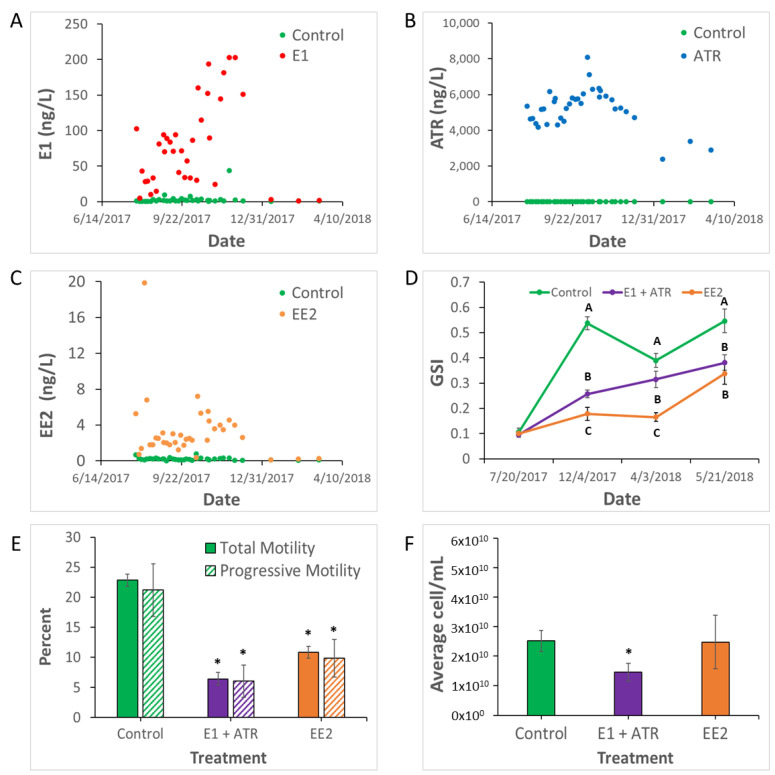
Summary of water chemistry and male largemouth bass (LMB; *Micropterus salmoides*) reproductive condition: (**A**,**B**) concentrations of estrone (E1; green = concentration in control pond, red = concentration in dosed ponds) and atrazine (ATR; green = concentration in control pond, blue = concentration in dosed ponds) as measured in the mixture and control ponds at each weekly sampling and averaged across ponds (n = 3); (**C**) concentration of 17alpha-ethinylestradiol (EE2) at each weekly sampling across ponds and averaged across ponds (n = 3); (**D**) mean gonadosomatic index (GSI; ± standard error) of male LMB at the four timepoints (see Table S1 for n), different letters denote significant differences (*p* < 0.05); (**E**) percent total sperm motility (solid bars) and percent progressive sperm motility (hashed bars) at the April time point (Control n = 12, E1 + ATR n = 11, EE2 n = 15), just prior to spawning, asterisks denote significant differences compared to control (*p* < 0.05); (**F**) sperm count (cell count per mL milt) at the April time point (Control n = 12, E1 + ATR n = 11, EE2 n = 10), just prior to spawning, asterisk denotes a significant difference compared to control (*p* < 0.05).

The average concentrations of chemicals in the outdoor experimental ponds were monitored weekly throughout the exposure. Mean water concentrations in the treatment ponds were E1 (47.9 ng/L), ATR (5.4 µg/L), and EE2 (2.4 ng/L) (Figure 1A–C). Dosing was stopped in December, and within a month the average concentration of EE2 was 0.1 ng/L and E1 was 2.9 ng/L. However, ATR continued to persist at an average of 2.9 µg/L throughout the remainder of the study (May). Water quality parameters varied seasonally, but there were no significant differences among the different experimental ponds. All raw water quality, water chemistry, and fish metric data for this experiment can be found at https://doi.org/10.5066/P9U2U3A1 (accessed on 11 December 2022) [26].

### 2.2. Reproductive Condition

GSI was significantly reduced in E1 + ATR (*p* < 0.05) and EE2 (*p* < 0.003) exposed males compared to control at every time point after early gonad recrudescence (December, April, and May; Figure 1D).

Sperm motility (percent total and progressive motility per sample) assessed at the April sampling timepoint was significantly reduced in both the E1 + ATR (*p* < 0.006) and EE2 (*p* < 0.02) treatments compared to control (Figure 1E). A decline in sperm count was also observed in the E1 + ATR (*p* < 0.03) treatment compared to control (Figure 1F). Sperm motility raw data can be found at https://doi.org/10.5066/P9M2WOJO (accessed on 11 December 2022) [27].

## 3. Discussion

Alterations in reproductive condition were observed in adult male LMB exposed to both chemical treatments. GSI values in the control group fell within normal ranges observed during a recrudescence and reproductive cycle in LMB [1,18,28]. Average GSI in the males exposed to the mixture of E1 + ATR or EE2 was significantly lower than the males in the control group, and this effect persisted throughout the spawning cycle. Reduced GSI from exposure to exogenous estrogens and environmental contaminants has been shown to have implications on testis development and male reproductive readiness and also has been correlated with reduced fertility [22,29,30].

Sperm motility and sperm count were significantly reduced with exposure to E1 + ATR. Similar trends have been observed in adult fathead minnows (*Pimephales promelas*) exposed to ATR alone [29]. Across various species of fish, EE2 has been shown to significantly reduce GSI, sperm count, and sperm motility [30,31,32]. The mechanisms by which estrogens act to impair male reproductive condition are not well understood [23]. It is hypothesized that exogenous estrogens can alter steroidogenic gene expression reducing circulating androgens, affecting gonad maturation and gamete production [23]. EE2 and the natural steroid 17beta-estradiol (β-E2) are among the most potent chemicals in terms of binding and activating the primary estrogen receptor, Erα, in vertebrates [33]. E1 does not have high binding affinity to the estrogen receptor across vertebrates, but has been shown to be readily converted to β-E2 in vivo leading to endocrine disruption in fish [13]. The mixture treatment containing E1 in the current study resulted in reduced male reproductive condition for all endpoints measured. Although sperm count was not significantly reduced in the EE2 treatment compared to control in the current study, the significant reduction of GSI and sperm motility indicates reduced reproductive capacity in these males. Sperm quality parameters, including sperm production (cell count) and function (motility) in male fish have been identified as key determinants of fertility [23,34]. Thus, the individual level physiological effects observed in the present study suggest that population level effects at the studied exposure concentrations are possible.

Exposure to estrogens and ATR during a sensitive window can lead to latent generational effects. Cleary et al. showed that medaka (*Oryzias latipes*) exposed to either EE2 or ATR during gamete development with no significant alterations in sperm parameters observed in the F0 generation, had significant reductions in sperm count and motility in the F2 generation [35]. In the current study, E1 concentrations rapidly degraded to concentrations comparable to control ponds within weeks of exposures ending (Figure 1A). However, environmentally relevant concentrations of ATR remained in the ponds after exposures ended and persisted through spawning (Figure 1B). Therefore, effects on reproductive condition in the E1 + ATR treatment could be latent effects from the E1 exposure, continued effects of the persistent ATR, or a combined effect of both. Fish exposure to EE2 only occurred during early recrudescence because treatment was halted and the hormone rapidly degraded as the experiment proceeded (Figure 1C), indicating that the estrogen induced effects persisted even after the exposure stopped. Although the concentrations in this study were comparable to those observed in the environment, the design represents a worst-case scenario with sustained concentrations for an extended period of time covering all of early gonad recrudescence. A more realistic exposure scenario would incorporate pulsed exposure mimicking increases in contaminant concentration from runoff during rain events [9,11]. Shorter duration exposures could pinpoint even further the timing during recrudescence that is most sensitive to effects of endocrine-disrupting compounds. The data in the current study indicate overt reproductive toxicity with the mixture or estrogen exposures. These observations warrant further investigation upon potential transgenerational and population level effects, in addition to the more real-world pulse exposure scenarios.

Taken together, our data indicate that environmentally observed concentrations of E1 + ATR or EE2 only can contribute to reduced reproductive condition and function in adult male LMB. Reduced GSI, sperm motility, and sperm counts have implications for the reproductive readiness and fertility of these male LMB [36]. Early recrudescence appears to be a sensitive window in the adult male LMB reproductive cycle, and effects of exposure during recrudescence persisted throughout the following spawning cycle. The mechanisms of action and potential population-level consequences of reproductive effects of exposures to these environmental endocrine disruptors still require further investigation.

## 4. Materials and Methods

### 4.1. Exposure and Animal Care

Pond exposures, sample processing, and data analysis were conducted at the U.S. Geological Survey Columbia Environmental Research Center (CERC, Columbia, MO, USA), and the sperm motility analysis was performed at the U.S. Geological Survey Wetland and Aquatic Research Center (WARC, Lafayette, LA, USA) after tissue shipment overnight (Supplementary Material). This study complied with all applicable sections of the Final Rules of the Animal Welfare Act regulations (nine CFR) and all CERC Institutional Animal Care and Use Committee guidelines for the humane treatment of the test organisms during culture and experimentation.

The exposure was conducted in nine 50′ × 72′ (0.08 acres) experimental ponds containing approximately 110,000 gallons of water. These ponds were filled with well water prior to the study, and conditions were maintained within criteria set forth by the American Standards and Testing Materials (ASTM 2004) for toxicity testing with aquatic organisms. General water quality averaged 200 mg/L hardness, 165 mg/L alkalinity, 8.1 pH, 0.0300 mg/L ammonia throughout the study. Alkalinity, hardness, pH, and ammonia were monitored weekly. Dissolved oxygen and temperature were measured twice a week. Detailed water quality testing methods can be found in Supplementary Material.

Adult LMB (three to five years old) were stocked into the ponds in April prior to the start of the study allowing approximately three months of acclimation prior to the start of chemical exposure. All fish were distributed in the ponds so that each pond contained 35 fish of similar length and weight. There was no difference in the body condition between ponds. These ponds were then randomly assigned treatments, and the treatments were in triplicate, so there were three ponds for each treatment (control, EE2, or E1 + ATR). These are open pond systems with naturally occurring populations of tadpoles/frogs, crayfish, invertebrate species, algae, etc. In addition to the LMB, five similar size one-to-two-year-old grass carp (*Ctenopharyngodon idella*) that had been spawned at CERC were stocked in each pond to help with maintenance of algae and vegetation. All ponds were fed equal amounts of pellet food throughout the study (no difference in feed rate between treatments) to supplement the naturally occurring prey sources in the ponds.

### 4.2. Dosing and Water Chemical Analysis

The analytes measured in the pond water were parent compounds 17alpha-ethinylestradiol, estrone, and Atrazine, Metabolites and breakdown products were not measured. Dosing solutions were confirmed by LC/MS, and pond concentrations were monitored weekly during the exposure period (July to December) and monthly once dosing stopped. The analytes (17alpha-ethinylestradiol, estrone, Atrazine, >98% purity) were purchased from Sigma–Aldrich (St. Louis, MO, USA). Carbon-13 internal standards were acquired from Cambridge Isotope Laboratories in solution (2,3,4-13C3-estrone [13C3-E1] and 20,21-13C2- ethynylestradiol [13C2-EE2], >98% purity) at 100 µg/mL in methanol. The solvents (acetonitrile, methanol, and water) were LCMS grade, and the ammonium hydroxide (28% in water) were from Fisher Scientific (Hampton, VA, USA).

Pond water samples were collected in 250 mL solvent rinsed, amber glass bottles, and 200 mL of controls or sample were immediately filtered through glass fiber filters (Whatman 0.7 µm GF/F, Fisher Scientific) using an SPE vacuum manifold (Biotage, Uppsala, Sweden). Water samples were then extracted via C18 SPE cartridges (Strata C18-E, 1 g, 20 mL, Phenomenex, Torrance, CA, USA) and analyzed via LC/MS using a previously published method (See Supplementary Material) [37].

To achieve the desired nominal concentrations in the ponds and maintain these concentrations while accounting for degradation, pond water samples were collected and analyzed weekly. Those concentrations were used to estimate how much degradation was occurring and what concentration should be used for dosing the treatment ponds the following week. The ponds were dosed weekly with either ethanol only for the solvent control ponds and those ponds that did not need additional chemical, or they were dosed with a calculated amount of stock solution (in ethanol) to bring the total pond concentration back to the desired nominal concentration of that treatment. Ethanol alone or chemicals in ethanol were added to the ponds at a maximum of 0.0001% (1 ppm) ethanol in the pond water at the time of dosing. The dosing stock solutions were added to the center of the ponds near an aerator, and the water mixing was accomplished by the movement created by the aerator. This dosing occurred from mid-July through November, with no further dosing in December.

### 4.3. Fish Collection and Processing

At the July and December sampling timepoints six fish were seined from each pond. Our target was three males and three females from each pond. However, externally determining sex is not possible this time of the year, so sex was not determined until dissection. Fish were sampled until three of each sex or four of one sex was reached, as we did not want to skew the sex ratio for the later timepoints. During the April sampling, four males and four females from each pond were brought into the lab for dissection. Sex was externally determined and then confirmed with dissection. At the May sampling, all remaining fish from each treatment were collected. Sample numbers for the males collected from each timepoint can be found in the Supplementary Material. Fish were euthanized with an overdose (300 mg/L) of buffered MS-222 (Ethyl 3-aminobenzoate methanesulfonate, Sigma–Aldrich) for fish metric measurements and tissue sample collection. Fish were blotted dry with a paper towel and weight (±0.01 g) was then measured on an electronic balance (Ohaus, Parsippany, NJ, USA), and total length was measured (cm). Gonads were excised with forceps and dissecting scissors, and then the tissue was weighed (±0.01 g). Gonad weight was used to calculate GSI (total gonad weight/total fish weight × 100). At the pre-spawning timepoint in April when mature sperm would be present, a posterior section of testis was collected, and an aliquot of milt was extracted from the remaining testis.

### 4.4. Sperm Count Analysis

Milt was collected immediately following weighing by pipetting 1 µL from the posterior end of the excised testis once the section for motility analysis was removed. The hemocytometer was prepared with coverslip in place. Preserved LMB milt in 4% paraformaldehyde (100-fold dilution) was diluted further in a second 1:100 dilution in phosphate buffered saline and gently mixed. A 10 µL aliquot was added to each side of the hemocytometer and incubated at room temperature for 5 min. The hemocytometer was viewed at 200X magnification on a Nikon 90i microscope (Minato City, Japan) and NIS Elements AR software (Nikon; version 4.10.01) was used to count cells in 2 of the 16 grid corner areas on each side (a total of 4–16 grids or 64 squares). This number was then back calculated to determine the average number of cells per mL milt for each treatment.

### 4.5. Sperm Motility Analysis

Cells were collected from a 1 mg piece of the posterior section of testis from each male that had been shipped in Hank’s Balanced Salt Solution at 308 mOsm/kg on wet ice packs overnight from CERC to WARC. Milt from the shipped tissue (3 µL) was activated with 40 µL top water, then 3 µL of the activated mixture was placed in a chambered slide (Leja Products, Nieuw-Vennep, The Netherlands; cat. no. 20 SC20-010040-B) and immediately analyzed by computer-assisted sperm motion analysis (CASA). Samples were viewed by using an Axio microscope (Zeiss, Jena, Germany) at 200X magnification, with data being collected on an average total of 408 sperm cells per replicate sample, with one or two replicates assessed per individual. Total and progressive motility, as well as other parameters, were analyzed with SpermVision v.3.9 software (MOFA Global, Veron, MI, USA) by using a digital camera (1MB-12FT, IMI Technology Co., LTD, Encinitas, CA, USA) operating at 60 frames per second (See Supplementary Material for camera settings) [22]. Data are presented as percent total and percent progressive motility. Percent total motility was evaluated as number motile sperm cells per total sperm cells analyzed in each sample. Percent progressive motility was evaluated as the number of sperm cells with forward, straight motion per total sperm cells analyzed. Replicate readings of the same sample were averaged before statistical analysis was performed to determine treatment effects.

### 4.6. Statistics

All statistics were conducted with JMP 14.2.0 (SAS Institute Inc, Cary, NC, USA). A linear mixed effects model was used to determine there was no random effect of pond. Because of this and the low power from small sample sizes, ponds were pooled for analysis of treatment effects for all endpoints. The data did not have normal distribution, so a paired Wilcoxon method test was used.

Raw data can be found through the U.S. Geological Survey and is publicly available at https://doi.org/10.5066/P9U2U3A1 (accessed on 11 December 2022) [26] and https://doi.org/10.5066/P9M2WOJO (accessed on 11 December 2022) [27].

## Data Availability

Raw data can be found through the U.S. Geological Survey and is publicly available at https://doi.org/10.5066/P9U2U3A1 (accessed on 11 December 2022) and https://doi.org/10.5066/P9M2WOJO (accessed on 11 December 2022).

## Supplementary Materials

The following supporting information can be downloaded at: https://www.mdpi.com/article/10.3390/ijms232416131/s1.
