# Supplementary material for: Declines in Reproductive Condition of Male Largemouth Bass (Micropterus salmoides) Following Seasonal Exposure to Estrogenic Endocrine-Disrupting Compounds
Source: Int J Mol Sci. 2022 Dec 17;23(24):16131. doi: 10.3390/ijms232416131 (PMC9785829; doi:10.3390/ijms232416131)
Supplement: Supplementary file 1 [file ijms-23-16131-s001.zip › ijms-1975635-supplementary.pdf]

Declines in reproductive condition of male largemouth bass (*Micropterus salmoides*) following seasonal exposure to estrogenic endocrine disrupting compounds

Jessica K. Leet [1,*], Catherine A. Richter [1], Robert W. Gale [1], Donald E. Tillitt [1] and Jill A. Jenkins [2]

[1] U.S. Geological Survey, Columbia Environmental Research Center, Columbia, MO, USA 1;
[2] U.S. Geological Survey, Wetland and Aquatics Research Center, Lafayette, LA, USA;
* Correspondence: jleet@usgs.gov; Tel.: +1 573-441-2784

Water Chemical Analysis

Pond water samples were collected in 250 mL solvent-rinsed, amber glass bottles, and 200 mL of controls or sample were immediately filtered through glass fiber filters (Whatman 0.7 µm GF/F, Fisher Scientific) using an SPE vacuum manifold (Biotage). Either 200 mL or 250 mL of filtered water was amended with 1% methanol and then spiked with 12.5 ng labeled surrogate (13C2-EE2). End-capped C18 SPE cartridges (Strata C18-E, 1 g, 20 mL, Phenomenex) were conditioned with 10 mL methanol, then equilibrated with 10 mL water. The water samples were loaded onto the SPE cartridge at about 5-10 mL/minute; the cartridges were washed with 10 mL of 15% methanol/water and dried under vacuum for 5-10 minutes. The analytes (estrone, E1; atrazine, ATR; 17alpha-ethinylestradiol, EE2) were eluted from the SPE cartridges with two 6-mL portions of methanol. The extracts were evaporated to dryness under nitrogen (N-Evap), reconstituted first, with 125 µL methanol, then diluted to 500 µL with 375 µL water. Final extracts were spiked with 12.5 ng labeled instrumental internal standard (13C3-E1).

Separations were performed by an Acquity Series H ultrahigh performance liquid chromatograph (Waters). Sample volumes of 10 µL were injected onto an Acquity BEH C18 column (1.7 µm x 2.1 x 50 mm) with a VanGuard precolumn (1.7 µm 2.1 mm) from Waters.

The initial mobile phase system was comprised of 0.1% ammonium hydroxide in water (A) and methanol (B). Gradient elution was performed by starting with a mobile phase composition to 25% B that was held from for 2.50 min, the mobile phase was linearly ramped to 95% B over 10.00 min, and held for 2.50 min, before returning to initial conditions and equilibrating by ramping to 10% B over 0.10 min. and holding for 2.40 min. Slowly increasing back-pressure

from this gradient system prompted changing to a less viscous, acetonitrile-based mobile phase system. This second gradient system was comprised of 0.1% ammonium hydroxide in water (A) and acetonitrile (B). Gradient elution was performed by starting with a mobile phase composition to 10% B held from for 2.50 min, the mobile phase was linearly ramped to 95% B over 10.00 min, and held for 2.50 min, before returning to initial conditions and equilibrating by ramping to 10% B over 0.10 min. and holding for 2.40 min. For both mobile phase systems, the flow rate was 400 µL/min and the column temperature was 40.0 °C. The first 2.40 min the column eluate was diverted to waste, then flow was redirected to the mass spectrometer until the completion of the gradient (17.50 min).

Analytes were detected using a Xevo TQ-XS tandem-quadrupole mass spectrometer (Waters) interfaced with an electrospray-ionization source operated in negative polarity and controlled using MassLynx (v 4.2). Compound-dependent mass spectrometry parameters were selected by directly infusing analyte and internal-standard solutions at concentrations of approximately 1.0 µg/mL into the mass spectrometer at a flow rate of 5.0 µL/min. Electrospray conditions were optimized by adjusting each parameter to maximize analyte signal-to-noise ratios. The optimized electrospray parameters were as follows: a capillary potential of 2.30 kV, cone voltage 19 V, source offset 30.0 V, Source temperature 150 °C, desolvation temperature 650 °C, cone gas flow 200 L/h, desolvation gas flow 1,200 L/h, nebulizer 7.00 bar, Argon collision gas flow was 0.20 mL/min.

Data were acquired in scheduled multiple-reaction-monitoring mode with a single acquisition window of from 2.50-17.50 min and a scan time of 0.40 s. The acquisition collected data for MRM transitions monitoring the primary (quantification) and secondary (confirmation) product ions. Identification and quantification of analytes required retention times within ±0.05 min of the retention time of the associated calibration standards; the analyte peak was required to contain both the primary and secondary product ions; and the ratio of the two product ions must have been within 30% when compared with calibration standards. Calibration standards ranged from 0.05 to 100 ng/mL, starting at the analyte detection limit. Analyte responses in standards and samples were normalized to the associated 13C-labeled analog using the internal standard method (isotope dilution). Quantification was performed using a 1/x weighted regression and all analyte regressions had a coefficient of determination (R2) of greater than 0.99.

A twelve-point calibration curve (0.05 to 100 ng/mL; 25 ng/mL IS) of each component was run with each analytical set, with calibration standards interspersed between every four samples. The limit of detection (LOD) for each analyte was determined as three times the standard deviation of the lowest-level calibration standard. LOD values for each analyte were: 0.02 ng/mL extract, or 0.02 ng/L dilutor water. Analyte recoveries typically ranged from 75-110%.

Water Metrics:

Temperature and DO

A YSI PRO20 meter was calibrated per manufacturer's instructions before each use. The probe was moved gently in the pond water with the probe completely covered while the reading stabilized, then the reading was recorded.

Ammonia

Total Ammonia was measured on a Hach DR3900 photometer using Hach TNT Plus 830 Ammonia vials and ammonia standard (Hach 189149) per manufacturer's instructions.

pH

Measurements of pH were taken using a glass electrode probe on a Thermo Orion 320 pH meter using a National Bureau of Standards scale[1]. The meter was calibrated prior to each use. Both calibration and measurements were conducted per manufacturer's instructions.

(1) Durst, R. Standard Reference Material: Standardization of pH measurements. National Bureau of Standards Special Publication: 1975.

Alkalinity

Alkalinity measurements were based on titration of a water sample using sulfuric acid to a pH of 4.5 measured on a Thermo Orion 320 pH meter. The volume of sulfuric acid needed to reach a pH of 4.5 is used to determine alkalinity. Titration was performed after pH was measured; the probe was left in the sample on the stir plate. A Kimble automatic burette was used for the titration. The burette was filled with 0.2N sulfuric acid and placed over the sample and titrated into the sample until the pH read 4.5. Then the difference in the volume was determined to calculate what volume of acid had been added. This acid volume was used to determine the alkalinity by a conversion table. The alkalinity is reported in mg $CaCO_3$ per milliliter of water.

Hardness

Alkalinity measurements were based on titration of a water sample using ethylenediaminetetraacetic acid (EDTA) until a colorimetric reaction occurred and the sample turned blue. A very small amount of Eriochrome black T powder (Sigma 858390) was added to the sample used to measure alkalinity. Then about 2 mL of ammonium hydroxide buffer was added to turn the sample bright pink. A Kimble automatic burette was filled with EDTA and placed over the sample then titrated into the sample until it turned blue. The volume of EDTA needed to achieve the blue color change was used to determine the hardness by a conversion table. The hardness is reported in mg CaCO3 per milliliter of water.

Sperm Motility

Software settings were: area of cell identification was 6 by 30 μm^2, cells classified as immotile were distance straight line < 1.0 micron. Cells with velocity curved line > 80 micron per second, linearity < than 0.65 (velocity straight line/by velocity curved line µm/s), and amplitude of lateral head displacement = 6.5 microns were classified as hyperactive, and those with distance average path/by radius > 3.0 microns and linearity < 0.5 microns were curvilinear. Progressive motility is forward motion; total motility includes all measurable types of motion, including vibratory movement, circular, non-progressive and progressive motion.

Any use of trade, firm, or product names is for descriptive purposes only and does not imply endorsement by the U.S. Government.

Table S1. Adult largemouth bass sample sizes (n) collected per timepoint per treatment

	Control		E1 + ATR		EE2	
Sampling Timepoint	Pond ID	n	Pond ID	n	Pond ID	n
July	9	3	16	3	10	2
	14	4	21	3	15	2
	20	3	23	4	17	1
	Total	**10**	Total	**10**	Total	**5**
December	9	4	16	4	10	4
	14	4	21	4	15	4
	20	4	23	2	17	4
	Total	**12**	Total	**10**	Total	**12**
April	9	4	16	3	10	5
	14	4	21	4	15	5
	20	4	23	4	17	5
	Total	**12**	Total	**11**	Total	**15**
May	9	4	16	4	10	4
	14	4	21	4	15	5
	20	4	23	4	17	4
	Total	**12**	Total	**12**	Total	**13**

E1 = estrone; ATR = atrazine; EE2 = 17alpha-ethinylestradiol
